# HDAC6 Interacts With Poly (GA) and Modulates its Accumulation in c9FTD/ALS

**DOI:** 10.3389/fcell.2021.809942

**Published:** 2022-01-12

**Authors:** Giulia del Rosso, Yari Carlomagno, Tiffany W. Todd, Caroline Y. Jones, Mercedes Prudencio, Lillian M. Daughrity, Mei Yue, Karen Jansen-West, Jimei Tong, Wei Shao, Yanwei Wu, Monica Castanedes-Casey, Lilia Tabassian, Björn Oskarsson, Karen Ling, Frank Rigo, Dennis W. Dickson, Tso-Pang Yao, Leonard Petrucelli, Casey N. Cook, Yong Jie Zhang

**Affiliations:** ^1^ Department of Neuroscience, Mayo Clinic, Jacksonville, FL, United States; ^2^ Neuroscience Graduate Program, Mayo Clinic Graduate School of Biomedical Sciences, Jacksonville, FL, United States; ^3^ Department of Neurology, Mayo Clinic, Jacksonville, FL, United States; ^4^ Ionis Pharmaceuticals, Carlsbad, CA, United States; ^5^ Department of Pharmacology and Cancer Biology, Duke University School of Medicine, Durham, NC, United States

**Keywords:** HDAC6, C9orf72, frontotemporal dementia, amyotrophic lateral sclerosis, dipeptide repeat proteins

## Abstract

The aberrant translation of a repeat expansion in chromosome 9 open reading frame 72 (*C9orf72*), the most common cause of frontotemporal dementia (FTD) and amyotrophic lateral sclerosis (ALS), results in the accumulation of toxic dipeptide repeat (DPR) proteins in the central nervous system We have found that, among the sense DPR proteins, HDAC6 specifically interacts with the poly (GA) and co-localizes with inclusions in both patient tissue and a mouse model of this disease (c9FTD/ALS). Overexpression of HDAC6 increased poly (GA) levels in cultured cells independently of HDAC6 deacetylase activity, suggesting that HDAC6 can modulate poly (GA) pathology through a mechanism that depends upon their physical interaction. Moreover, decreasing HDAC6 expression by stereotaxic injection of antisense oligonucleotides significantly reduced the number of poly (GA) inclusions in c9FTD/ALS mice. These findings suggest that pharmacologically reducing HDAC6 levels could be of therapeutic value in c9FTD/ALS.

## Introduction

Frontotemporal dementia (FTD), caused by frontotemporal lobar degeneration (FTLD) in the brain, can be characterized by a range of clinical symptoms, including dementia, language impairment, and changes in personality and behavior. Amyotrophic lateral sclerosis (ALS) is caused by the degeneration ofi motor neurons and is characterized by a progressive loss of motor control. FTD and ALS share several genetic and pathologic features and are considered part of the same disease spectrum. As the most common genetic cause of both FTD and ALS, cases with G_4_C_2_ hexanucleotide repeat expansions in the *C9orf72* gene are collectively referred to as c9FTD/ALS. In addition to the aggregation of TAR DNA-binding protein 43 (TDP-43), protein inclusions containing dipeptide repeat (DPR) proteins translated from the sense (G_4_C_2_) and antisense (C_4_G_2_) repeat expansion are also detected in c9FTD/ALS patients. Specifically, repeat associated non-ATG (RAN) translation drives production of poly (GA), poly (GR) and poly (GP) proteins from sense G_4_C_2_ repeat RNA, and poly (PA), poly (PR) and poly (GP) proteins from antisense G_2_C_4_ repeat RNA (Mori et al., 2013a; Ash et al., 2013; Mori et al., 2013b; Gendron et al., 2013; Zu et al., 2013). The levels of sense-derived DPRs are higher than antisense-derived DPRs, with poly (GA)-positive inclusions being the most abundant DPR protein pathology (Mackenzie et al., 2014; Mackenzie et al., 2015).

In order to identify new therapeutic strategies for FTD and ALS, recent studies have explored the use of antisense oligonucleotides (ASOs) or pharmacologic inhibitors of histone deacetylase 6 (HDAC6) to rescue axonal transport defects in induced pluripotent stem cell (iPSC)-derived motor neurons from familial ALS patients (Guo et al., 2017; Fazal et al., 2021). Notably, HDAC6 inhibition was able to increase mitochondrial motility in the axon (Guo et al., 2017; Fazal et al., 2021) and rescue TDP-43 pathology (Fazal et al., 2021), as well as restore neurite outgrowth and neuromuscular junction morphology in a co-culture system composed of iPSC-derived motor neurons and primary mesoangioblast-derived myotubes (Stoklund Dittlau et al., 2021). These findings suggest that HDAC6 inhibition and/or knockdown could be protective in FTD and ALS, but the potential involvement of HDAC6 in c9FTD/ALS pathogenesis has yet to be specifically evaluated.

Given that markers of the ubiquitin proteasome system (including ubiquitin and p62) have been shown to co-localize with DPR protein pathology in c9FTD/ALS patients (Mori et al., 2013a; Ash et al., 2013; Mori et al., 2013b; Gendron et al., 2013; Zu et al., 2013), and that HDAC6 has a high affinity for ubiquitin (Boyault et al., 2006), we evaluated whether HDAC6 co-localizes with DPR protein inclusions in c9FTD/ALS. We found that the presence of HDAC6-positive inclusions differentiates c9FTD/ALS from sporadic FTD/ALS cases, and also observed that, among the sense DPR proteins, HDAC6 specifically interacts with poly (GA). Furthermore, HDAC6 overexpression increased poly (GA) levels in cells expressing 66-mer G_4_C_2_ repeats. Interestingly, this increase was dependent on HDAC6 expression, but not on its deacetylase activity. Finally, we show that HDAC6 also localizes to DPR protein inclusions in a mouse model of c9FTD/ALS, and we observed a significant reduction in poly (GA) pathology in mice following treatment with ASOs targeting HDAC6. Collectively, these findings suggest that HDAC6 interacts with poly (GA) and potentiates poly (GA) aggregation, and further indicate that strategies to reduce HDAC6 expression may represent a novel therapeutic avenue for c9FTD/ALS.

## Materials and Methods

### Human Tissues

Post-mortem hippocampal and frontal cortical tissues from control, FTD or ALS patients (including sporadic and familial cases with the *C9orf72* repeat expansion) were obtained from the Mayo Clinic Florida Brain Bank. Information on human patients is provided in Supplementary Table S1. Written informed consent was obtained before study entry from all subjects or their legal next of kin if they were unable to give written consent, and biological samples were obtained with Mayo Clinic Institutional Review Board (IRB) approval.

### Immunohistochemistry in Human and Mouse Brain

The tissue sections were deparaffinized in xylene, rehydrated in a graded series of alcohols, and washed in dH2O. Antigen retrieval was performed by steaming slides in dH2O, citrate buffer/pH6 for 30 min, and endogenous peroxidase activity was blocked by incubation in Dako peroxidase Block (S2001, DAKO). To detect HDAC6 or the sense DPR proteins, sections were immunostained with primary antibody (Supplementary Table S2) using the DAKO Autostainer (Universal Staining System) and the DAKO EnVision + HRP system. Following labeling, all sections were counterstained with hematoxylin (Thermo Fisher Scientific), dehydrated through ethanol and xylene washes, and coverslipped with Cytoseal mounting medium (Thermo Fisher Scientific). Slides were scanned with a ScanScope® AT2 (Leica Biosystems), and representative images were taken with ImageScope® software (v12.1; Leica Biosystems).

### Immunofluorescence Staining in Human and Mouse Brains

Paraffin sections (5 μm) of human and mouse brain tissues were deparaffinized, rehydrated, steamed for 30 min in Dako antigen retrieval solution, blocked with Dako All Purpose Blocker for 1 h, and incubated with primary antibody (Supplementary Table S2). After washing, sections were incubated with corresponding Alexa Fluor 488-, 568- or 647-conjugated donkey anti-species (1:500, Molecular Probes) for 2 h. Hoechst 33258 (1 μg/ml, Thermo Fisher Scientific) was used to stain cellular nuclei. Images were obtained on a Zeiss LSM 880 laser scanning confocal microscope.

### Quantification of Neuropathology

To quantify the percent of HDAC6 inclusions positive for poly (GA), poly (GP) and poly (GR), images were captured by confocal microscopy. The total number of HDAC6 inclusions (∼22–130 per patient), as well as the number of inclusions that were also positive for each DPR, were counted in a blinded fashion in the hippocampus of c9FTD/ALS patients.

To quantify HDAC6 and DPR pathology, high resolution digitized images of immunostained mid-sagittal serial sections were obtained by using a ScanScope AT2 (Leica Biosystems). For HDAC6, the cortex was annotated and the number of inclusions in this region was quantified manually in a blinded fashion. For DPR pathology, the hippocampus was annotated and quantitative analysis of DPR immunoreactivity was performed using a custom-designed color deconvolution algorithm and ImageScope software (Aperio, Vista, CA). As previously described (Janocko et al., 2012), the algorithm was designed to measure the optical density of the brown chromogen as a percentage of burden within an annotated region of interest.

### Generation of Plasmid Constructs

The generation of the GFP-tagged DPR expression constructs and the GFP control has been previously described (Zhang et al., 2014). A plasmid containing human wild-type HDAC6 in a pCMV-SPORT6 vector was purchased from Life Technologies, and the QuikChange Mutagenesis Kit (Agilent Technologies, Clara, CA, United States) was utilized to generate a catalytically inactive HDAC6 mutant (H216/611A) following the manufacturer’s protocol. Wild-type and H216/611A HDAC6 were cloned into a mycHis-pcDNA3.1 vector using HindIII and NotI restriction sites. All constructs were sequence-verified prior to use.

### Cell Culture and Transient Transfections

HEK293T cells were maintained in Opti-Mem (Life Technologies) supplemented with 10% heat-inactivated FBS (Life Technologies) and 1% penicillin-streptomycin (Life Technologies) and passaged every 3–4 days based on 90% confluency. Cells grown in 6-well plates were transfected with the indicated plasmids using Lipofectamine 2000 (Thermo Fisher Scientific) and harvested 24–48 h post-transfection.

### Preparation of Cell Lysates

Cell pellets were lysed in co-immunoprecipitation (co-IP) buffer (50 mM Tris–HCl, pH 7.4, 300 mM NaCl, 1% Triton X-100, 5 mM EDTA) plus 2% SDS and both protease and phosphatase inhibitors, sonicated on ice, and then centrifuged at 16,000 × g for 20 min. Supernatants were saved as cell lysates. The protein concentration of lysates was determined by BCA assay (Thermo Fisher Scientific), and samples were then subjected to Western blot and immunoassay analyses.

### Co-Immunoprecipitation

Co-immunoprecipitation assays were performed as previously described (Zhang et al., 2009; Zhang et al., 2016). In brief, HEK293T cells grown in 6-well plates were transfected for 48 h with expression vectors encoding Myc-tagged HDAC6_WT_ with GFP, GFP-(GA)_50_, GFP-(GP)_47_ or GFP-(GR)_50_. Cell pellets were lysed in co-IP buffer containing protease and phosphatase inhibitors. After sonication on ice, the lysates were centrifuged at 16,000 x g for 20 min and the protein concentration of the resulting supernatants was determined by BCA assay. Supernatants containing 500 μg of total protein were precleared with 15 μL Dynabeads Protein G (Life Technologies) and then incubated with a mouse polyclonal anti-Myc antibody (MA1-980, 1:250, Invitrogen) overnight at 4°C with gentle shaking. The antigen-antibody immuno-complex was captured by Dynabeads Protein G for 4 h and then the beads were separated using a magnetic tube stand. After washing 3 times with co-IP buffer, captured proteins were eluted from the beads using loading buffer and samples were subjected to Western blot analysis.

### Western Blot Analysis

Cell lysates were diluted with 2x SDS-loading buffer at a 1:1 ratio (v/v) and then heated at 95°C for 5 min. Afterwards, equal amounts of protein were loaded into 10-well 4–20% Tris-glycine gels (Novex). After transferring proteins to PVDF membranes, non-specific sites were blocked with 5% nonfat dry milk in TBS plus 0.1% Tween 20 for 1 h and then incubated with primary antibody (Supplementary Table S2) overnight at 4°C. Membranes were washed in TBST and incubated with donkey anti-rabbit or anti-mouse IgG antibodies conjugated to horseradish peroxidase (1:5000; Jackson ImmunoResearch) for 1 h. Protein expression was visualized by enhanced chemiluminescence treatment and exposure to film. Bands were quantified using Scion Image by analyzing pixel density, and protein levels were normalized to GAPDH as the protein loading control.

### Immunoassay Analysis of Poly (GA) and Poly(GP)

To detect and quantify poly (GP) and poly (GA) proteins in cell lysates, we used a previously described sandwich immunoassay using Meso Scale Discovery (MSD) electrochemiluminescence detection technology (Su et al., 2014; Gendron et al., 2015). To quantify poly (GP), we coated MSD Multi-array 96-well plates (Cat#L15XA-6, MSD) with a polyclonal anti-poly (GP) antibody, and to detect HA-tagged poly (GA) protein, we coated the plates with anti-HA clone 12CA5 (Roche, 11583816001). Wells were washed with TBS-T (0.2% Tween 20 in TBS) and blocked with 3% milk [poly (GP)] or 3% MSD Blocker A (Cat#R93AA-1, MSD) [poly (GA)] in TBS-T at room temperature for 1 h, then washed 3 times with TBS-T. For both DPR protein assays, 20 µg of sample was added to wells in duplicate and plates were then incubated for 2 h at room temperature at 600 rpm and washed 3 times with TBS-T. For detection of poly (GP) proteins, an anti-poly (GP) polyclonal antibody and a Sulfo-Tag Goat Anti-Mouse Antibody (Meso Scale Discovery, R32AC-5) was added to the wells. To detect poly (GA) proteins, an anti-poly (GA) antibody was conjugated to SULFO-TAG NHS-Ester group according to the manufacturer’s recommendation (Cat#R91AO-1, MSD). The plates were incubated for 1 h at room temperature at 600 rpm, after which time the plates were again washed 3 times with TBS-T and read with 1X MSD Read Buffer T with Surfactant (Cat#R92TC-2, MSD) on the MSD Meso QuikPlex SQ 120 Plate Reader. Response values corresponding to the intensity of emitted light upon electrochemical stimulation of the assay plate were background corrected using the average response from lysates obtained from non-transfected cells.

### Animal Studies

All procedures using mice were performed in accordance with the National Institutes of Health Guide for Care and Use of Experimental Animals and approved by the Mayo Clinic Institutional Animal Care and Use Committee (IACUC).

### Virus Production

Recombinant adeno-associated virus (AAV) 9 was produced as previously described (Chew et al., 2015; Chew et al., 2019). Briefly, AAV vectors expressing (G_4_C_2_)_2_ or (G_4_C_2_)_66_ were co-transfected with helper plasmids in HEK293T cells using polyethylenimine (23966, Polysciences, Inc.). Cells were harvested 48 h following transfection and lysed in the presence of 0.5% sodium deoxycholate and 50 units/mL benzonase (Sigma-Aldrich) by freeze-thawing. The virus was isolated using a discontinuous iodixanol gradient. The genomic titer of each virus was determined by quantitative reverse transcriptase PCR (qRT-PCR), and AAV solutions were diluted in sterile phosphate-buffered saline (PBS).

### Neonatal Viral Injections

Intracerebroventricular (ICV) injections of virus were performed as previously described (Chew et al., 2015; Chew et al., 2019). Briefly, 2 µL (1×10^10^ genomes/µl) of AAV-(G_4_C_2_)_2_ or AAV-(G_4_C_2_)_66_ solution was manually injected into each lateral ventricle of cryoanesthetized C57BL/6J mouse pups on postnatal day 0 (P0). Pups were allowed to recover from cryoanesthesia on a heating pad and were then returned to the home cage with their mother.

### Antisense Oligonucleotide Injections

Control and HDAC6 ASOs were developed and provided by ionis Pharmaceuticals. The sequence for the control ASO (GTT​TTC​AAA​TAC​ACC​TTC​AT) was screened against endogenous mouse genes to reduce the probability of impacting expression. The control ASO was a 20-nucleotide MOE-gaper ASO, wherein the central gap segment was comprised of ten 2′-deoxyribonucleotides flanked on the 5 and 3′ wings by five 2′-O-methoxyethyl (MOE) modified nucleotides. The HDAC6 ASO (GCC​TAC​TCT​TTC​GCT​GTC) was an 18 nucleotide MOE-gaper ASO, in which the central gap segment was comprised of eight 2′-deoxyribonucleotides flanked on the 5 and 3′ wings by five MOE modified nucleotides. Control or HDAC6 ASO was injected into the central nervous system (CNS) of 3-month-old AAV-(G_4_C_2_)_2_ or AAV-(G_4_C_2_)_66_ mice by means of stereotactic ICV injection, as previously described (Gendron et al., 2017; Cook et al., 2020) with some minor modifications. Specifically, 10 μL of control or HDAC6 ASO solution (corresponding to 500 μg ASOs) was delivered into the right lateral ventricle using the coordinates: 0 mm anterior and 1.0 mm lateral to the right of the bregma and 1.9–2.0 mm deep as measured from the brain surface.

### Tissue Processing

For downstream protein, immunostaining and RNA analyses of brain tissue, mice were euthanized by CO_2_. The brain was rapidly removed and hemisected. Sagittal half brains were immersion fixed in 4% paraformaldehyde and embedded in paraffin, and 5 µm sections were mounted on glass slides for immunofluorescence or immunohistochemistry staining. The other half brain was separated into forebrain and hindbrain and rapidly frozen.

### Preparation of Brain Lysates

Frozen forebrain samples were weighed and homogenized in 5x volume TE buffer (50 mM Tris base (pH7.4), 50 mM NaCl, 1 mM EDTA, 1 mM PMSF, 1x protease and phosphatase inhibitor cocktails). For RNA extraction, 90 µL of homogenate was added to 270 µL Trizol LS (Life Technologies, Carlsbad, CA), and the mixture was frozen at -80°C until extraction was performed as described below. To obtain the SDS-soluble protein fraction, 90 µL of homogenate was added to 110 µL of co-immunoprecipitation buffer (50 mM Tris–HCl, pH 7.4, 300 mM NaCl, 1% Triton X-100, 5 mM EDTA) with 2% SDS and protease and phosphatase inhibitor cocktails. Following a brief sonication, lysates were centrifuged at 16,000 × g for 20 min, and a BCA assay was performed on the supernatant to determine protein concentration.

### RNA Extraction, Reverse Transcription and qPCR

Total RNA was extracted from the forebrain using the Direct-zol RNA MiniPrep kit (Zymo Research) according to manufacturer’s instructions. cDNA was then obtained following reverse transcription of 250 ng of the extracted RNA with random primers and the High Capacity cDNA Transcription Kit (Applied Biosciences). To quantify RNA levels of the indicated transcripts in mouse brain or cultured cells, qRT-PCR was conducted in triplicate for all samples using a SYBR green assay (Thermo Fisher Scientific) on an ABI Prism 7900HT Fast Real-Time PCR System (Applied Biosystems). Primer sequences are listed in Supplementary Table S3. Relative RNA expression of *Hdac6* and *C9-66R* was normalized to *Gapdh* or *GAPDH* values as an endogenous transcript control.

### Data Analyses

All statistical analyses were performed in GraphPad Prism. Data are presented as mean ± SEM. Data was analyzed by two-tailed unpaired t test or one-way ANOVA followed by *Tukey’s post-hoc* analysis. *p* < 0.05 was considered statistically significant.

## Results

### HDAC6 Co-localizes With DPR Pathology in c9FTD/ALS

**FIGURE 1:**
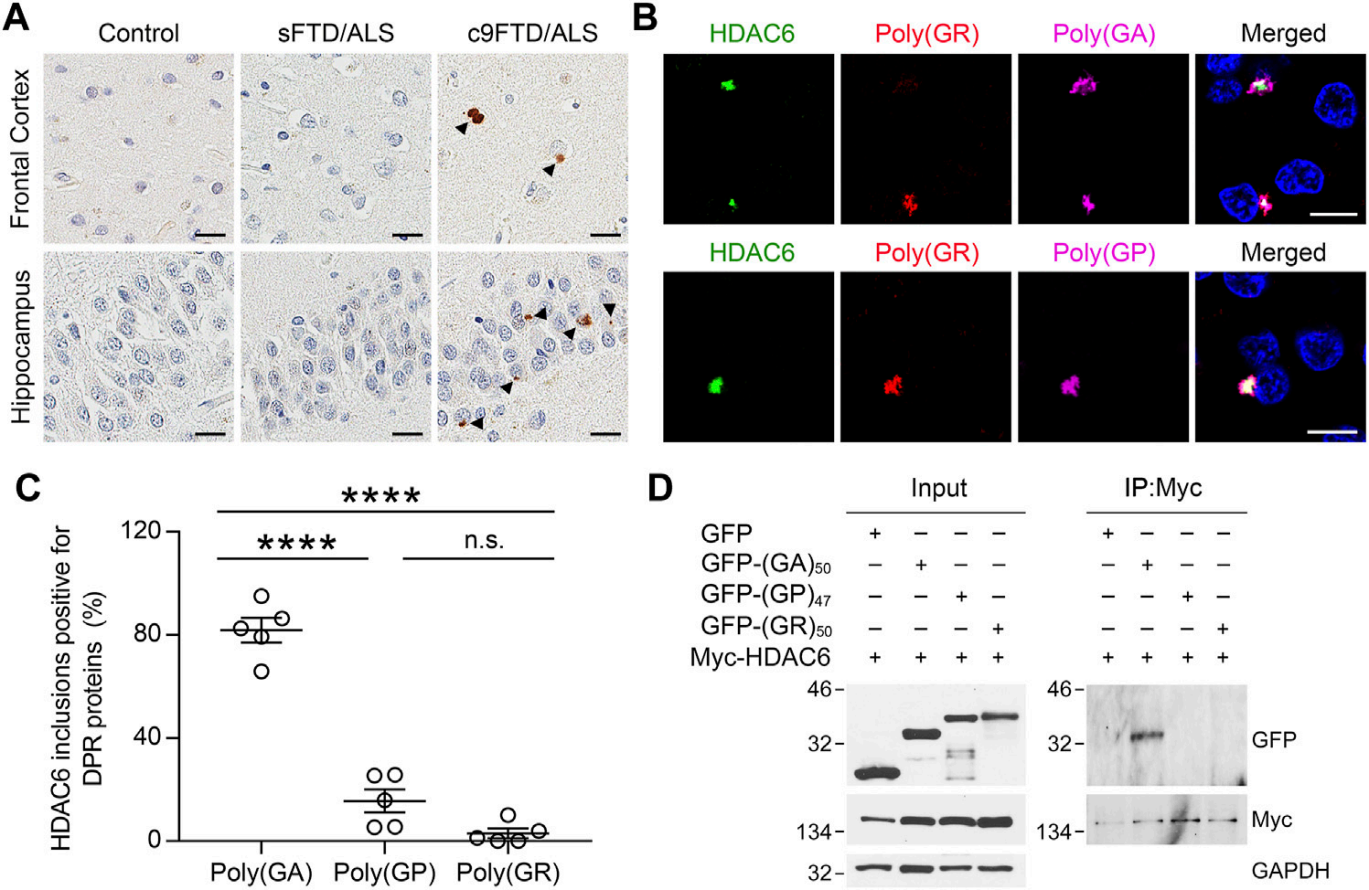
HDAC6 co-localizes with DPR pathology in c9FTD/ALS. **(A)** Representative images of immunohistochemical analysis of HDAC6 in frontal cortex (top panels) and hippocampus (bottom panels) from control, sporadic FTD/ALS, or c9FTD/ALS patients (*n* = 5 per group). Arrowheads indicate HDAC6 inclusions. Scale bars, 20 μm. **(B)** Triple-immunofluorescence staining for HDAC6, poly (GR), and either poly (GA) (top panel) or poly (GP) (bottom panel) in the hippocampus of c9FTD/ALS patients. Scale bars, 10 μm. **(C)** Quantitative analysis of the percentage of HDAC6-positive inclusions that co-localize with poly (GA), poly (GP) and poly (GR) in the hippocampus of c9FTD/ALS patients (*n* = 5). Data are presented as mean ± SEM, ****p < 0.0001, one-way ANOVA, Tukey’s multiple-comparison test. **(D)** HEK293T cells were transfected with myc-HDAC6 and GFP, GFP-(GA)_50_, or GFP-(GP)_47_. GFP and myc-HDAC6 levels were evaluated in cell lysates by immunoblotting (left panel), with GAPDH used to control for protein loading. Myc-HDAC6 or GFP were immunoprecipitated from cell lysates, followed by immunoblotting for myc or GFP (right panel).

Given that HDAC6 co-aggregates with ubiquitin and α-synuclein in Parkinson’s disease (Kawaguchi et al., 2003), and elevated HDAC6 expression has been reported in FTLD patients with TDP-43 pathology (Odagiri et al., 2013), we wanted to examine the pattern of HDAC6 immunoreactivity in affected regions of the brain in both sporadic (sFTD/ALS) and c9FTD/ALS patients. Notably, HDAC6-positive inclusions were detected in the frontal cortex and hippocampus of c9FTD/ALS patients, but were absent from both sFTD/ALS patients and healthy controls (Figure 1A). Since the presence of DPR protein pathology differentiates c9FTD/ALS from sFTD/ALS, we examined whether the HDAC6-positive inclusions co-localized with the most abundant DPR proteins, namely poly (GA), poly (GP), and poly (GR). We observed that HDAC6 co-localized with all three DPR proteins in inclusions (Figure 1B). Quantitative analysis revealed that there was significantly greater co-localization between HDAC6-positive inclusions and poly (GA) compared to poly (GP) or poly (GR) (Figure 1C).

To examine whether the presence of HDAC6 inclusions in c9FTD/ALS might be due to an interaction between HDAC6 and DPR proteins, HEK293T cells expressing Myc-tagged HDAC6 were co-transfected with green fluorescent protein (GFP) or GFP-tagged DPR repeat constructs, including GFP-(GA)_50_, GFP-(GP)_47_, and GFP-(GR)_50_. We used a myc antibody to immunoprecipitate myc-HDAC6, confirming efficient immunoprecipitation in all cell lysates by Western blotting. Consistent with the co-localization between HDAC6 and poly (GA) in c9FTD/ALS patients, GFP-(GA)_50_ co-immunoprecipitated with myc-HDAC6, while GFP-(GP)_47_ and GFP-(GR)_50_ did not, despite a similar level of GFP expression in all cell lysates (Figure 1D). These results indicate that, among the sense DPR proteins, HDAC6 specifically interacts with poly (GA).

### HDAC6 Potentiates Poly (GA) Accumulation

**FIGURE 2:**
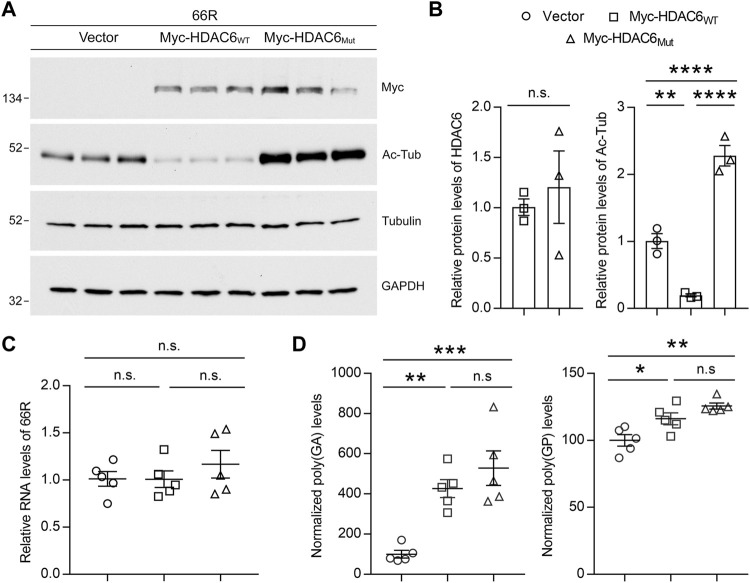
Overexpression of HDAC6 increases poly (GA) levels. **(A,B)** Immunoblots **(A)** and densitometric analysis of immunoblots **(B)** for the indicated proteins to examine the levels of acetylated tubulin (Ac-Tub) in HEK293T cells co-expressing 66R with vector, HDAC6_WT_ or HDAC6_Mut_ (*n* = 3 independent experiments). Data are presented as mean ± SEM. In **(B)**, (left) n.s. not significant, two-tailed unpaired t test (right) **p < 0.01, ****p < 0.0001, one-way ANOVA, Tukey’s multiple-comparison test. **(C)** qRT-PCR was performed to assess 66R mRNA levels in HEK293T cells co-expressing 66R with vector, HDAC6_WT_ or HDAC6_Mut_ (*n* = 5 independent experiments). Data are presented as mean ± SEM, n.s. not significant, one-way ANOVA, Tukey’s multiple-comparison test. **(D)** Poly (GA) and poly (GP) levels in HEK293T cell lysates were measured by immunoassay (*n* = 5 independent experiments). Data are presented as mean ± SEM, n.s. not significant; *p < 0.05, **p < 0.01, ****p < 0.0001, one-way ANOVA, Tukey’s multiple-comparison test.

To assess whether changes in HDAC6 expression or activity impact DPR protein levels, we co-transfected (G_4_C_2_)_66_ (66R) with Myc-tagged wild-type HDAC6 (Myc-HDAC6_WT_) or a catalytically inactive mutant (Myc-HDAC6_Mut_) in cultured cells. We first confirmed that both the wild-type and catalytically inactive HDAC6 mutant were expressed at equal levels (Figures 2A,B). Using the deacetylation of tubulin as an indicator of HDAC6 activity, we also demonstrated that wild-type HDAC6 significantly reduced tubulin acetylation, while the catalytically inactive HDAC6 mutant significantly increased acetylated-tubulin levels in a dominant-negative manner (Figures 2A,B). We then verified that neither changes in HDAC6 expression nor changes in its activity impacted G_4_C_2_ repeat RNA levels by qRT-PCR (Figure 2C). Finally, we examined the abundance of poly (GA) and poly (GP) proteins by Meso Scale Discovery (MSD) immunoassays, which revealed that both wild-type and catalytically inactive HDAC6 increased poly (GA) levels by over fourfold, while they only slightly increased poly (GP) levels (Figure 2D). These results indicate that HDAC6 preferentially potentiates the accumulation of poly (GA) independently of its deacetylase activity.

### Reduction of HDAC6 Mitigates Poly (GA) Pathology

**FIGURE 3:**
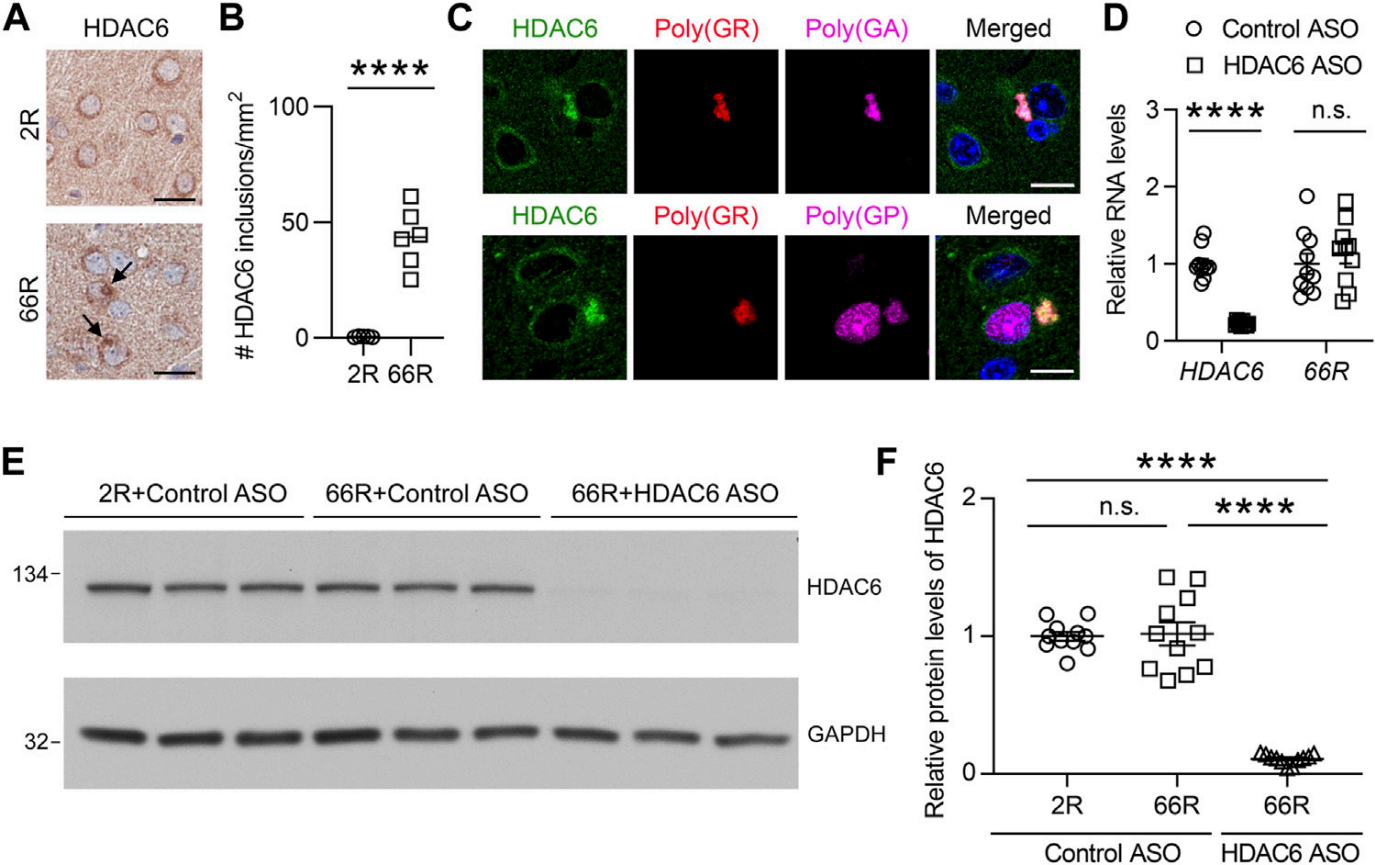
HDAC6 ASO reduces HDAC6 levels in a c9FTD/ALS mouse model. **(A)** Representative images of immunohistochemical analysis of HDAC6 in the cortex of mice injected with AAV-(G_4_C_2_)_2_ (2R) or AAV-(G_4_C_2_)_66_ (66R). Arrows indicate HDAC6 inclusions. Scale bars, 20 μm. **(B)** Quantification of the number of HDAC6 inclusions in 2R (*n* = 5) and 66R (*n* = 6) mice. **(C)** Triple-immunofluorescence staining for HDAC6, poly (GR), and poly (GA) in 66R mice. Scale bars, 10 μm. **(D)** qRT-PCR was performed to assess human *HDAC6* or 66R mRNA levels in 66R mice injected with either control ASO or HDAC6 ASO (*n* = 10 per group). Data are presented as mean ± SEM, n.s. not significant; ****p < 0.0001, two-tailed unpaired t test. **(E,F)** Immunoblots **(E)** and densitometric analysis of immunoblots **(F)** for the indicated proteins to examine the protein levels of HDAC6 in 2 and 66R mice injected with control ASO or HDAC6 ASO (*n* = 11 per group). Data are presented as mean ± SEM, n.s. not significant; ****p < 0.0001, one-way ANOVA, Tukey’s multiple-comparison test.

**FIGURE 4:**
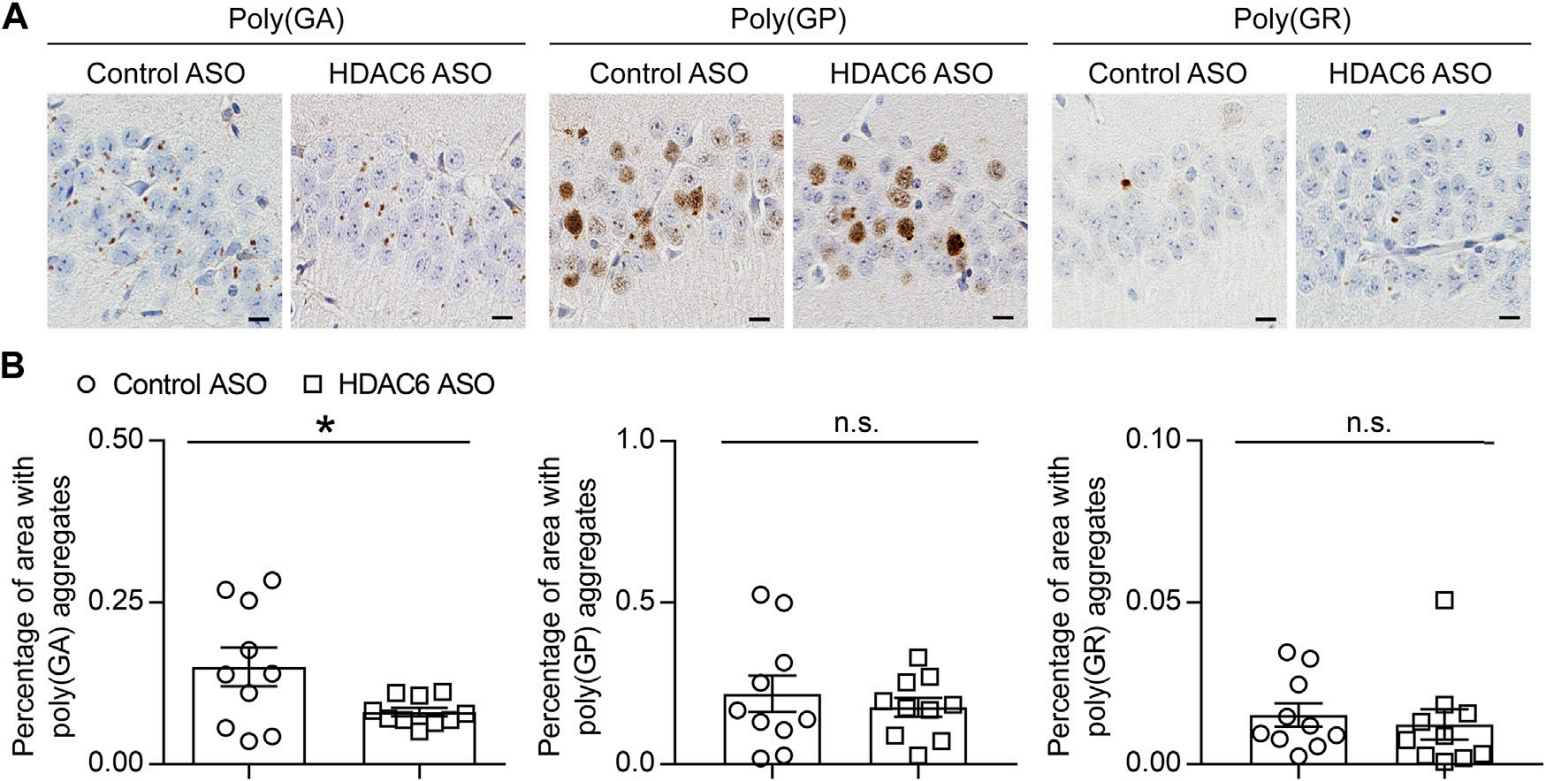
HDAC6 knockdown reduces poly (GA) pathology in a c9FTD/ALS mouse model. **(A)** Representative images of immunohistochemical analysis of poly (GA), poly (GP) and poly (GR) in the hippocampus of 66R mice injected with either control ASO or HDAC6 ASO. Scale bars, 20 μm. **(B)** Quantitative analysis of the percentage of poly (GA), poly (GP) and poly (GR) burden in the hippocampus of 66R mice injected with either control ASO or HDAC6 ASO (*n* = 11 per group). Data are presented as mean ± SEM, n.s. not significant; *p < 0.05, two-tailed unpaired t test.

We previously generated a mouse model of c9FTD/ALS by using somatic brain transgenesis to deliver adeno-associated virus (AAV) vectors that drive the expression of a *C9orf72*-G_4_C_2_ repeat expansion throughout the central nervous system (CNS) of mice. This model develops repeat length-dependent c9FTD/ALS-related behavioral and pathological abnormalities by 6 months of age, including abundant DPR protein pathology (Chew et al., 2015; Chew et al., 2019). We examined HDAC6 expression in these animals, and similar to c9FTD/ALS patients, we observed abnormal accumulation of HDAC6-positive inclusions in mice expressing the expanded G_4_C_2_ repeat (66R) but not in control mice (2R) (Figures 3A,B). Moreover, we observed that endogenous mouse HDAC6 also co-localized with DPR protein pathology in our c9FTD/ALS mouse model (Figure 3C). Based on these findings, we wondered whether reducing HDAC6 levels could alleviate DPR protein pathology. To test this idea, 3-month-old 66R mice were injected with ASOs targeting HDAC6 or a non-targeting control and harvested 3 months post-injection at 6 months of age. We first confirmed that HDAC6 ASOs efficiently reduced HDAC6 mRNA and protein expression (Figures 3D–F), while having no effect on G_4_C_2_ repeat-containing RNA levels (Figure 3D). We then performed immunohistochemistry to examine poly (GA), poly (GP), and poly (GR) pathology in the brain (Figure 4A). Following quantification, we observed a significant reduction in poly (GA) burden in the hippocampus of HDAC6 ASO-treated animals (Figure 4B). In contrast, no differences in poly (GP) or poly (GR) burden were observed between control and HDAC6 ASO-treated groups (Figure 4B). These results are consistent with our finding that, among the sense DPR proteins, HDAC6 specifically interacts with poly (GA), and suggest that HDAC6 represents a novel therapeutic target to alleviate poly (GA) pathology in c9FTD/ALS.

## Discussion

The extent to which DPR protein accumulation contributes to the pathogenicity of the *C9orf72* repeat expansion is still under investigation, with most studies focusing on poly (GA), poly (GR) or poly (PR). Although less toxic than the arginine-containing DPRs (Mizielinska et al., 2014; Freibaum et al., 2015), poly (GA) aggregation has been linked to ER stress (Zhang et al., 2014), nucleocytoplasmic transport defects (Zhang et al., 2016), and ubiquitin proteasome system (UPS) impairment (Zhang et al., 2014; Yamakawa et al., 2015; Zhang et al., 2016). Expression of poly (GA) alone induced mild toxicity in lower model organisms (Mizielinska et al., 2014; Ohki et al., 2017) and resulted in neurodegeneration, gliosis, and FTD/ALS-like behavioral defects in mice (Zhang et al., 2016; Schludi et al., 2017). Furthermore, even though poly (GA) may not be the most toxic DPR, poly (GA) inclusions are the most abundant aberrant pathology observed in c9FTD/ALS patient brains (Mori et al., 2013b; Zhang et al., 2014; Mackenzie et al., 2015). Targeting poly (GA) with a specific antibody proved beneficial in a mouse model of c9FTD/ALS, although in this case, reducing poly (GA) aggregate levels also impacted the accumulation of other DPRs (Nguyen et al., 2020).

HDAC6 is unique in that it is not only associated with histone deacetylation, but is also a primarily cytoplasmic protein with several different substrates, most notably tubulin (Li et al., 2013; Simoes-Pires et al., 2013). Its role in regulating microtubule dynamics makes HDAC6 important for cell migration and synapse formation (Li et al., 2013; Simoes-Pires et al., 2013), as well as aggresome formation (Kawaguchi et al., 2003) and stress granule dynamics (Kwon et al., 2007). These latter functions rely not only on the deacetylase activity of HDAC6, but also on its C-terminal ubiquitin-binding zinc finger domain (Kawaguchi et al., 2003; Kwon et al., 2007). In fact, HDAC6 has one of the highest known affinities for ubiquitin (Seigneurin-Berny et al., 2001). We discovered that the effects of HDAC6 on poly (GA) accumulation are independent of its deacetylase activity, suggesting that perhaps its ubiquitin-binding activity dictates its association with poly (GA) inclusions. Indeed, poly (GA) inclusions are positive for ubiquitin, and cryo-electron tomography revealed that these aggregates also recruit an abundance of 26S proteasome complexes (Guo et al., 2018).

It remains unclear how HDAC6 regulates poly (GA) inclusion formation in c9FTD/ALS. Given that HDAC6 plays a critical role in regulating aggresome formation through binding to both polyubiquitinated misfolded proteins and dynein motors (Kawaguchi et al., 2003), it is possible that the HDAC6: poly (GA) interaction leads to enhanced poly (GA) accumulation and aggregation through the aggresome pathway. This could account for why reducing HDAC6 mitigates poly (GA) aggregation. Our observation that reducing HDAC6 levels modulates the accumulation of poly (GA) in mice further suggests that HDAC6 knockdown could be beneficial in c9FTD/ALS, and that HDAC6 could be an interesting therapeutic target. HDAC6 knockout mice are viable (Zhang et al., 2008), suggesting that a loss of HDAC6 activity is not overly detrimental *in vivo*, and HDAC6 inhibition also appears beneficial in several disease conditions (Simoes-Pires et al., 2013). It is worth noting that the therapeutic advantage of HDAC6-targeting ASOs may be limited, as reducing HDAC6 levels had little to no effect on the expression of the other DPR proteins, or on the levels of repeat-containing RNA. Nevertheless, it could be advantageous to combine agents that reduce HDAC6 expression levels with therapies that target other pathological features of c9FTD/ALS. Such combinatorial approaches warrant further investigation.

In conclusion, we have demonstrated that, among the sense DPR proteins, HDAC6 specifically interacts with poly (GA). HDAC6 also accumulates into DPR inclusions in both c9FTD/ALS patient tissue and a G_4_C_2_ repeat-expressing mouse model. Overexpression of HDAC6 increased poly (GA) levels in cultured cells independent of its deacetylase activity, indicating that HDAC6 expression mediates poly (GA) accumulation via a mechanism that likely relies upon the ubiquitin-binding domain of HDAC6. In addition, decreasing HDAC6 levels reduced poly (GA) accumulation in a c9FTD/ALS mouse model, suggesting that lowering HDAC6 levels could be of therapeutic value in c9FTD/ALS, especially when combined with other therapeutic approaches.

## Data Availability

The original contributions presented in the study are included in the article/Supplementary Material, further inquiries can be directed to the corresponding authors.

## Abbreviations

ALS, amyotrophic lateral sclerosis; C9orf72, chromosome 9 open reading frame 72; CNS, central nervous system; DPR, dipeptide repeat; FTD, frontotemporal dementia; FTLD, frontotemporal lobar degeneration; HDAC6, histone deacetylase 6; RAN, repeat-associated non-canonical; TDP-43, TAR DNA-binding protein 43.
