# Supplementary material for: HDAC6 Interacts With Poly (GA) and Modulates its Accumulation in c9FTD/ALS
Source: Front Cell Dev Biol. 2022 Jan 12;9:809942. doi: 10.3389/fcell.2021.809942 (PMC8790530; doi:10.3389/fcell.2021.809942)
Supplement: Supplementary file 1 [file DataSheet1.doc]

| **Case #** | **Pathological Diagnosis** | **Gender** | **Age at Onset** | **Age at death** | **Disease**  **Duration** | ***C9orf72* repeat expansion** |
| --- | --- | --- | --- | --- | --- | --- |
| 1 | Normal | F | N/A | 60.0 | N/A | N/A |
| 2 | Normal | M | N/A | 74.8 | N/A | N/A |
| 3 | Control | M | N/A | 66.7 | N/A | N/A |
| 4 | VaD | F | N/A | 68.3 | N/A | N/A |
| 5 | Normal | F | N/A | 53.7 | N/A | No |
| 6 | ALS | F | 67.0 | 70.2 | 3.2 | No |
| 7 | ALS | F | 56.0 | 60.3 | 4.3 | No |
| 8 | FTLD/ALS | M | 66.3 | 67.3 | 1.0 | No |
| 9 | FTLD/ALS | M | 60.0 | 64.8 | 4.8 | No |
| 10 | ALS | F | 64.1 | 67.1 | 3.0 | No |
| 11 | FTLD/ALS | F | 53.3 | 60.3 | 7.0 | Yes |
| 12 | ALS | F | 65.5 | 68.1 | 2.6 | Yes |
| 13 | ALS | M | 56.7 | 62.0 | 5.3 | Yes |
| 14 | FTLD/ALS | M | 58.2 | 62.2 | 4.0 | Yes |
| 15 | FTLD | M | N/A | 80.2 | N/A | Yes |

**Table S1. Characteristics of patients with c9FTD/ALS**

FTLD, frontotemporal lobar degeneration; ALS, amyotrophic lateral sclerosis

N/A, Not Applicable

**Supplementary Table 2. Primary antibodies for Western blot, immunohistochemistry and immunofluorescence staining**

| **Western blot** |  |  |  |  |
| --- | --- | --- | --- | --- |
| **Antibody** | **Species** | **Dilution** | **Number** | **Company** |
| anti-GFP | rabbit | 1:4000 | A-6455 | Life Technologies |
| anti-Myc | mouse | 1:1000 | MA1-980 | Invitrogen |
| anti-GAPDH | mouse | 1:5000 | H86504M | Meridian Life Science |
| anti-HDAC6 | rabbit | 1:500 | 07-732 | EMD Millipore |
| anti-Acetyl-α-Tubulin | rabbit | 1:500 | 3971S | Cell Signaling |
| anti-α-tubulin | mouse | 1:1000 | T5168 | Sigma-Aldrich |
| **Immunohistochemistry** |  |  |  |  |
| **Antibody** | **Species** | **Dilution** | **Number** | **Company** |
| anti-GA | rabbit | 1:50000 | Rb9880^a^ |  |
| anti-GP | rabbit | 1:10000 | Rb5823^a^ |  |
| anti-GR | rabbit | 1:2500 | Rb7810^a^ |  |
| anti-HDAC6^*^ | rabbit | 1:500 | 07-732 | EMD Millipore |
| anti-HDAC6^b, #^ | rabbit | 1:100 |  |  |
| **Immunofluorescence** |  |  |  |  |
| **Antibody** | **Species** | **Dilution** | **Number** | **Company** |
| anti-HDAC6^*^ | rabbit | 1:500 | 07-732 | EMD Millipore |
| anti-HDAC6^b, #^ | rabbit | 1:100 |  |  |
| anti-GR | rat | 1:500 | MABN778 | EMD Millipore |
| anti-GA | mouse | 1:500 | MABN889 | EMD Millipore |
| anti-GP | mouse | 1:100 | TALS 828.66 | Target ALS Foundation |

^a^, Antibody described in: Gendron T. F. *et al.*, *Acta Neuropathol* **126**, 829-844 (2013).

^b^Antibody described in: Kawaguchi Y, et al., *Cell* **115(6)**,727-38 (2003).

*Anti-HDAC6 antibody was used for staining on human tissues.

^#^Anti-HDAC6 antibody was used for staining on mouse tissues.

**Table S3**. Primers for qPCR

| **Model** | **Target** | **Primers** |
| --- | --- | --- |
| mouse brain | *C9-66R* | 5’-TAGTACTCGCTGAGGGTGAAC-3’  5’-CTACAGGCTGCGGTTGTTTC-3’ |
| mouse brain | *Gapdh* | 5’-CATGGCCTTCCGTGTTCCTA -3’  5’-CCTGCTTCACCACCTTCTTGAT-3’ |
| mouse brain | *Hdac6* | 5’-CGCTGGAGGTCAGGACATGAA-3’  5’-TCTAGGCCTGCTGCAGGAATG-3’ |
| cultured HEK293T cells | *C9-66R* | 5’-tacagctcctgggcaacg-3’  5’-cttgttcaccctcagcgagt-3’ |
| cultured HEK293T cells | *GAPDH* | 5’-gttcgacagtcagccgcatc-3’  5’-ggaatttgccatgggtgga-3’ |
